# The Impact of Preoperative Opioid Use Disorder on Complications and Costs following Primary Total Hip and Knee Arthroplasty

**DOI:** 10.1155/2019/9319480

**Published:** 2019-12-18

**Authors:** Jacob M. Wilson, Kevin X. Farley, Matthew Aizpuru, Eric R. Wagner, Thomas L. Bradbury, George N. Guild

**Affiliations:** ^1^Department of Orthopaedic Surgery, Emory Orthopaedics & Spine Center, 59 S Executive Park NW, Atlanta, GA 30329, USA; ^2^Emory School of Medicine, 201 Dowman Dr., Atlanta, GA 30322, USA

## Abstract

**Introduction:**

Multiple studies have demonstrated that patients taking opioids in the preoperative period are at elevated risk for complications following total hip (THA) and knee (TKA) arthroplasty. However, the incidence and impact of opioid use disorder (OUD) among these patients—both clinically and fiscally—remain unknown. The purpose of this study was to investigate this relationship.

**Methods:**

The Nationwide Readmission Database (NRD) was used to identify patients undergoing THA and TKA from 2011 to 2015. Coarsened exact matching was used to statistically match the OUD and non-OUD cohorts. Further analysis was then conducted on matched cohorts with multivariate analysis. The incidence of OUD was also determined, and the costs associated with this comorbidity were calculated.

**Results:**

The incidence of OUD in arthroplasty patients increased 80% over the study period. OUD patients had higher odds of prosthetic joint infection (OR 1.55, 95% CI 1.23–1.94), wound complication (OR 1.40, 95% CI 1.12–1.76), prosthetic complication (OR 1.37, 95% CI 1.10–1.70), and revision surgery (OR 1.47, 95% CI 1.19–1.81). OUD patients also had longer length of stays (TKA: +0.67 days; THA: +1.09 days), higher readmission (OR 1.60, 95% CI 1.43–1.79), and increased 90-day costs (TKA: +$3,602 [95% CI $3,138–4,065]; THA: +4,527 [95% CI $3,593–4,920).

**Conclusion:**

Opioid use disorder is becoming a more common comorbidity among THA and TKA patients. This is concerning as it represents a significant risk factor for postoperative complication. It additionally confers increased perioperative costs. Patients with OUD should be counseled on their elevated risk, and future work will be needed to determine if this is a modifiable risk factor.

## 1. Introduction

The opioid epidemic in the United States is well documented. The United States has experienced an alarming number of drug overdose deaths, and the majority (63% in 2015) involve opioids [1]. This epidemic, not surprisingly, has garnered attention from the media, government, and medical practitioners. In 2017, the White House declared the opioid crisis a public health emergency. While many of the aforementioned overdoses are a result of illicit drug use, prescription opioid use also contributes significantly [2]. Estimates are that patients treated with opioids for chronic pain misuse their narcotics 21–29% of the time, and 8–12% of these patients are addicted to the prescribed drug [3].

In the adult reconstruction realm, prior research has demonstrated that patients prescribed preoperative opioids are at higher risk for perioperative morbidity, revision, and less satisfactory outcomes [4–11]. Much attention has also been paid to postoperative opioid regimens, and there has been increasing recognition that conservative postoperative prescription patterns of opioids can be both safe and effective [12]. However, there has been a substantial increase in the number of patients being prescribed long-term opioids for chronic pain in the community (estimated at a 3-4% of those treated for chronic pain) [13, 14]. While the majority of these prescriptions are not written by orthopedists [15], surgical candidates taking opioids preoperatively are frequently encountered.

Less common is the patient with a diagnosed opioid use disorder (OUD). This diagnosis is defined by the Diagnostic and Statistical Manual of Mental Disorders 5 (DSM-5) as a problematic pattern of opioid use leading to clinically significant impairment or distress. DSM-IV had previously classified the same disorder as opioid abuse or dependence, and this diagnosis is made when 2 of 11 diagnostic criteria are present in a 12-month period [16]. Much less is known about this patient population, and how frequently these patients are receiving total joint arthroplasty is unknown. Defining risk in these patients is critical, and in value-based reimbursement models, understanding the economic impact of this diagnosis is important.

Therefore, the purpose of this investigation was three-fold: (1) we sought to determine the incidence of opioid dependence or abuse in the total hip and knee arthroplasty population, (2) to determine if those with opioid dependence are at higher risk for postoperative complication, and (3) to determine the economic burden associated with this diagnosis. We hypothesized that the incidence of opioid dependence among total joint arthroplasty patients is increasing and that these patients would have higher complication rates and increased resource utilization.

## 2. Materials and Methods

### 2.1. Data Acquisition

Patients included in this study were selected from the Nationwide Readmission Database (NRD), a publicly available administrative database maintained by the Agency for Healthcare Research and Quality (AHRQ), a part of the Healthcare Cost and Utilization Project (HCUP). Information regarding this database can be found at https://www.hcup-us.ahrq.gov/tech_assist/centdist.jsp. The NRD includes information on patients of all payer types, including the uninsured. The database provides a nationally representative sample and is able to capture all readmissions occurring in the same state, including readmissions to hospitals different from where the index admission took place. The database contains data on the patient's initial inpatient stay, as well as all subsequent inpatient admissions occurring in the same calendar year. It can thus be queried for postoperative inpatient complication data, as well as diagnoses leading to readmission. This allows for the capture of major complications requiring readmission out to 90 days postoperatively.

Patients were identified using the International Classification of Disease, Ninth Revision, Clinical Modification (ICD-9-CM) procedural codes (total hip arthroplasty 81.51; total knee arthroplasty 81.54). The database was queried from 2011 to 2015 quarter 3 (Q3). This was chosen as the end date for this study as this was when the database switched to ICD-10 codes, and exclusion of this transition allowed for equal comparisons. The NRD cannot track patients from year to year, and therefore, we excluded all patients undergoing primary hip or knee arthroplasty in quarter 4 (Q4) of each year as complete 90-day follow-up could not be obtained in these Q4 patients.

ICD-9 diagnosis codes were used to identify patients with opioid use disorders (OUD; abuse or dependence), and the following codes were used: 304.00–304.03, 304.70–304.73, and 305.50–305.53. Similarly, postoperative complications were identified using ICD-9 codes. Hospital costs were calculated using NRD cost-to-charge ratios and were inflation adjusted to the year 2015 using the Consumer Price Index from the Federal Reserve Bank [17].

### 2.2. Baseline Patient Information

Baseline patient data were collected including the following variables: age, sex, surgery type (THA or TKA), insurance status, zip code income quartile, hospital size, comorbidities, and the presence or not of perioperative transfusion. The included comorbidities were obtained from the Elixhauser Comorbidity Measure [18] and included obesity, diabetes mellitus (DM), preoperative anemia, congestive heart failure (CHF), chronic kidney disease (CKD), chronic obstructive pulmonary disease (COPD), psychiatric illness, and tobacco use. Perioperative transfusion was included in this category as it is well established as a risk factor for postoperative infection [19].

### 2.3. Outcomes

The incidence of opioid use disorders among total hip and knee arthroplasty patients was calculated on an annual basis by dividing the number of patients with opioid use disorder by the total number of patients undergoing the procedure (with or without OUDs) annually. This was then compared between years. Additionally, we collected information on the following postoperative complications: prosthetic joint infection (PJI), superficial surgical site infection, other wound complications, prosthetic complications (including mechanical complication, loosening, dislocation, prosthetic fracture, and periprosthetic fracture), revision surgery, and postoperative pain (ICD-9 codes 338.18 and 338.19; acute postoperative pain). Complications were included in this study if they occurred on the initial inpatient stay or if they were the cause of readmission. We additionally collected length of stay, readmission, inpatient hospital costs, and 90-day episode costs for all included patients.

### 2.4. Statistical Analysis

After identification of all patients undergoing THA or TKA, the patients were then divided into two cohorts: patients with and without OUD. HCUP-provided weights were then used to create nationally representative estimates, and further analysis was then conducted. Ultimately, 3,438,105 patients were identified, and of these, 9,832 (0.29%) were found to have a concomitant diagnosis of OUD. First, analysis was conducted comparing baseline patient information between groups using chi-square analysis for categorical values and *t*-tests for continuous variables.

Given the differences identified between nonopioid dependent and OUD patients, statistical matching was performed using coarsened exact matching (CEM) to match patients based on a number of baseline factors. In short, CEM is a validated method of matching [20, 21] that temporarily coarsens data which then allows for exact matching. After matching is performed, typical multivariate analysis can then be conducted on matched cohorts. Patients were matched on all of the variables given in Table 1. While many were significantly different prior to matching, after matching was performed, no differences remained (*p* ≥ 0.997). Further analysis was then conducted on these matched cohorts.

Binomial logistic regression, controlling for the variables listed in Table 1, was conducted on matched cohorts to determine if a diagnosis of OUD was an independent predictor of postoperative complications, readmission, or nonhome discharge. Furthermore, cost and LOS were analyzed with linear regression. Additionally, linear regression was used to analyze the trend of incidence of OUD over time. A *p* value of <0.05 was considered significant. All statistics were performed using SPSS Statistics (IBM Corporation, Version 25, Armonk, NY).

## 3. Results

### 3.1. Baseline Patient Information and the Incidence of Opioid Use Disorder

Ultimately, 3,438,105 (THA: 35%, and TKA: 65%) patients were identified for inclusion in this study. 9,982 (0.29%) patients were identified as having a preexisting OUD (opioid abuse or dependency). Analysis comparing baseline patient information between patients with and without OUD was conducted prior to matching. This analysis revealed that opioid dependent patients included significantly more males, significantly more patients with Medicaid (and less with Medicare or private insurance), significantly younger patients, a higher rate of perioperative transfusion, a higher number of patients in lower income quartile zip codes, and a higher number of patients treated at high-volume centers, as well as significantly different comorbidity profiles (*p* < 0.001 for all comparisons) (Table 1).

**Figure 1:**
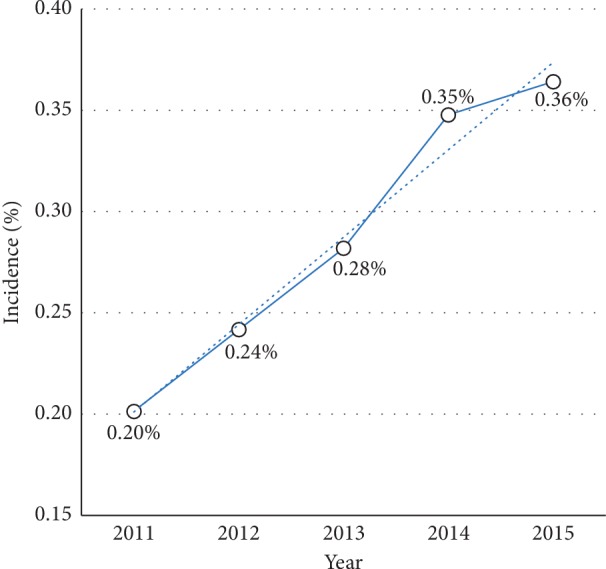
The annual incidence of opioid use disorder among patients undergoing total hip or knee arthroplasty in the United States from 2011 to 2015.

The incidence of opioid use disorder among those undergoing total hip or knee arthroplasty in the United States was determined by dividing the number of those with OUD each year by the total number of patients undergoing THA or TKA in the corresponding year. We found that the incidence of OUD in arthroplasty patients is increasing significantly, and from 2011 to 2015, the incidence nearly doubled from 0.2% to 0.36% (80% increase, *p* < 0.001) Figure 1.

### 3.2. Opioid Use Disorder and Postoperative Complications

Multivariate analysis conducted on matched cohorts found significant differences in the odds of multiple postoperative complications. These included prosthetic joint infection (OR 1.55, 95% CI 1.23–1.94, *p* < 0.001), superficial surgical site infection (OR 1.66, 95% CI 1.34–2.05, *p* < 0.001), noninfectious wound complication (OR 1.40, 95% CI 1.12–1.76, *p*=0.003), prosthetic complication (OR 1.37, 95% CI 1.10–1.70, *p*=0.005), need for early revision surgery (all cause revision; OR 1.47, 95% CI 1.19–1.81, *p* < 0.001), and for clinically important acute postoperative pain (OR 2.97, 95% CI 2.72–3.23, *p* < 0.001) (Table 2).

### 3.3. Length of Stay, Nonhome Discharge, 90-Day Readmission, and Economic Burden of Opioid Use Disorder

Multivariate analysis, again controlling for patient characteristics, was conducted to examine postoperative length of stay, nonhome discharge, and 90-day readmission. This analysis revealed that patients with OUD undergoing TKA and THA have significantly longer length of stays (TKA: +0.67 days; THA: +1.09 days, *p* < 0.001) as well as significantly higher odds of nonhome discharge (TKA: OR 1.47, 95% CI 1.30–1.64, *p* < 0.001; THA: OR 1.40, 95% CI 1.28–1.53, *p* < 0.001) and 90-day readmission (TKA: OR 1.60, 95% CI 1.43–1.79, *p* < 0.001; THA: OR 1.53, 95% CI 1.43–1.79, *p* < 0.001). We additionally compared both inpatient and 90-day costs between patients with and without OUD. This analysis revealed that OUD was associated with significantly increased inpatient (TKA: +$2,522 [95% CI $2,170–2,874]; THA: +2,924 [95% CI $2,496–3,353], *p* < 0.001) and total 90-day costs (TKA: +$3,602 [95% CI $3,138–4,065]; THA: +4,527 [95% CI $3,593–4,920], *p* < 0.001) in both TKA and THA (Table 3).

## 4. Discussion

The current investigation identifies a clear and concerning increase in the incidence of total hip and knee arthroplasty patients with concomitant opioid use disorders. This is troubling given that our study also demonstrates that this cohort of patients is at elevated perioperative risk for complications, increased length of stay, readmission, and resource utilization.

Our results demonstrated that the incidence of opioid use disorder among total hip and knee arthroplasty patients is increasing on an annual basis. This may be expected given the well-established opioid epidemic [13] along with chronic pain prescribing patterns [14]. A similar analysis conducted on pregnant women demonstrated similar findings with significantly increased rates of opioid use disorders over time [22]. The incidence of OUDs in patients undergoing lumbar fusion was found to be 0.91%, which is higher than our identified rate of 0.36% in 2015 [23]. This is also lower than the rate that was found in a similar study performed on patients undergoing lower extremity bypass (0.63%) [24] and lower than the 0.81% that has been reported for the general population [24, 25]. The lower rate observed in our cohort likely reflects the fact that patients undergoing total joint arthroplasty are typically older than the general population, and while substance abuse is becoming more common in a geriatric population [26], it remains much more commonly encountered in younger populations. This is also reflected in our two cohorts. Opioid dependent patients were, on average, younger than those without OUD. We controlled for this fact by matching patients on age and subsequently controlling for age in our multivariate regression.

Though novel, our finding that patients with OUD sustain high postoperative morbidity is consistent with prior literature. Similar analyses conducted on patients undergoing lumbar fusion [23] and lower extremity bypass [24] procedures demonstrated similar results, findings that patients with OUDs had higher rates of complications and nonhome discharge, as well as increased length of stay and total charges. Additionally, patients who are prescribed preoperative opioids before undergoing a THA or TKA are known to be at higher risk for postoperative complications and increased postoperative opioid consumption [4–7, 9–11, 27]. However, until now, analysis of patients with OUD has been limited to small series [28–30]. This population is likely very different than patients simply receiving opioids preoperatively for pain. In fact, the current study found that patients with opioid use disorder who undergo THA or TKA are on average, younger, from lower income zip codes, more likely to be smokers, and have higher comorbid burdens. These are important differences to consider given their impact on postoperative outcomes. While we took multiple steps (statistical matching and subsequently controlling for these differences in our statistical model) to control for these differences in our analysis, it should be recognized that patients with OUD are likely fundamentally different than those without OUD and the entirety of this difference may be difficult to fully account for. However, even when controlling for these differences and performing analysis on matched cohorts, rates of complication were significantly higher in the OUD cohort.

Infectious complications have previously been associated with substance abuse, as Bauer et al. found nearly a 40% infection rate in patients who illicitly use intravenous drugs after TKA [31]. Preoperative opioid use has been repeatedly associated with surgical site infections in arthroplasty [9, 11, 27] as well as other general surgery procedures [32, 33]. Why this may be the case is likely multifactorial and could include patient and socioeconomic factors. Nonetheless, some limited evidence exists to suggest that opioids are associated with delayed wound healing which could be partially responsible for some of our observed associations [34]. This is further supported by the finding that OUD patients had significantly higher rates of wound healing issues—both infectious and noninfectious. Direct impairment of immune cells by opioids has also been demonstrated in vitro and in animal models [35].

A significant finding of this investigation is that OUD represents an expensive comorbidity. As value-based models emerge, this is an important consideration and adjustments will need to be made for patients with OUD as they are at increased risk for readmission, complications, and subsequent increased costs. In this study, patients with OUD were significantly more costly. This was by over $2500 + for inpatient costs and $3600 + for total 90-day costs in both THA and TKA. These differences persisted, even when controlling for demographic, procedural, and comorbid data. Alternatively, we do not suggest that prior OUD is a contraindication to arthroplasty as prior studies demonstrate these patients do improve [6]. However, these patients must be recognized and counseled that they are at high risk, and when possible, should be weaned from narcotics preoperatively to reduce their risk of complications [9, 29].

There are multiple limitations to this investigation, most of which are inherent to analysis of large administrative databases. First, our analysis is reliant on accurate coding. It is possible that some patients with opioid abuse or dependence were not coded as such and were therefore included in the incorrect cohort. The same is true of the preoperative comorbidities included in our match and as controls in our multivariate model as well as our examined outcomes. On the same note, we assumed in our analysis that coding frequency stayed the same over time. Our observed increase in incidence, therefore, could be partially accounted for by an increased awareness of the opioid epidemic and increased coding for OUD over time. Similarly, the NRD reports only on patients who were treated as inpatients. Given the movement to outpatient total joint arthroplasty, this likely means that many patients were not captured. However, inclusion of patients treated as outpatients may have actually increased our observed differences between cohorts as these are generally healthy patients with low-risk profiles. Generally, opioid use disorder would be considered a contraindication to outpatient arthroplasty [36] and it, therefore, is likely that the vast majority of these patients have been captured in this investigation. Additionally, using the NRD, only inpatient complications are captured unless the complication leads to readmission and even readmissions are tracked only to 90 days. This could mean that our complication rates are underestimated. Last, it is true that complete isolation of OUD may be difficult. Still, this analysis was performed on matched cohorts and controlling for available comorbid, socioeconomic, and procedural data, likely providing the best analysis possible.

Despite its limitations, the use of the Nationwide Readmission Database is a strength of this study as it allows for national estimates and provides a very large, all-payer sample. It additionally allows for controlling of socioeconomic factors by reporting insurance status as well as zip code income quartile. Both factors that have been associated with outcomes [37–39], and the ability to control for these represents an important strength compared to other database studies. Also, readmissions can be tracked even if the readmission occurs at a different institution, therefore providing a higher capture rate for outcomes. Additionally, trends in incidence of OUD in the arthroplasty population could not confidently be reported without the use of a large administrative database, representing an advantage over single-institution studies. Finally, the NRD provides charge-cost ratios and reports economic data allowing for quantification of the additional cost conferred by OUD as a comorbid condition. As value-based reimbursement models emerge and evolve, this information is invaluable.

In conclusion, opioid use disorder as a preoperative comorbid condition is becoming increasingly common in the primary total hip and total knee arthroplasty patients. This is an important finding as this patient cohort has elevated risk for multiple important complications, readmission, extended length of stay, and increased economic burden in the early postoperative period. This patient population must be counseled on their elevated risks. Furthermore, this study adds to the evidence suggesting these patients should be weaned from preoperative narcotics, as opioid freedom prior to surgery, even after long-term use, confers a risk reduction [6, 7, 9, 27]. Future research may be directed at evaluating preoperative opioid use in morphine equivalents to determine cutoff values for safe surgery.

## Figures and Tables

**Table 1:** Patient demographics and comorbidities.

Characteristic		Nonopioid dependent (*n* (%))	Opioid dependent (*n* (%))	*p* value	
				Before matching	After matching
Total patients		3,428,273 (99.7)	9,832 (0.3)		

Surgery type	THA	1,198,470 (35)	4,258 (43.3)	<0.001	0.999
	TKA	2,229,803 (65)	5,574 (56.7)		

Sex	Male	1,347,632 (39.3)	4,693 (47.7)	<0.001	0.999
	Female	2,080,641 (60.7)	5,140 (52.3)		

Insurance	Medicare	1,941,276 (56.7)	4,576 (46.6)	<0.001	0.998
	Private	1,226,412 (35.8)	2,828 (28.8)		
	Medicaid	118,405 (3.5)	1,869 (19.0)		
	Other	137,350 (4.0)	552 (5.6)		

Age category	<55	486,664 (14.2)	3,550 (36.1)	<0.001	0.998
	55–64	984,362 (28.7)	3,852 (39.2)		
	65–74	1,191,172 (34.7)	1,810 (18.4)		
	75–84	653,333 (19.1)	540 (5.5)		
	85+	112,742 (3.3)	81 (0.8)		

Perioperative transfusion		374,279 (10.9)	1,463 (14.9)	<0.001	0.998

Zip code income quartile	1	728,949 (21.6)	2,348 (24.3)	<0.001	0.997
	2	861,933 (25.5)	2,602 (26.9)		
	3	900,568 (26.7)	2,523 (26.1)		
	4	886,595 (26.2)	2,189 (22.7)		

Hospital size	Low	741,092 (21.6)	1,278 (13.0)	<0.001	0.999
	Medium	866,705 (25.3)	2,596 (26.4)		
	High	1,820,476 (53.1)	5,958 (60.6)		

Comorbidity	Obesity	737,221 (21.5)	2,920 (29.7)	<0.001	0.999
	Diabetes	660,785 (19.3)	1,626 (16.5)	<0.001	0.999
	Psychiatric disorder	524,428 (15.3)	3,961 (40.3)	<0.001	0.998
	Anemia	490,946 (14.3)	2,046 (20.8)	<0.001	0.999
	CHF	87,133 (2.5)	335 (3.4)	<0.001	0.998
	Renal failure	165,033 (4.8)	562 (5.7)	<0.001	0.999
	COPD	507,748 (14.8)	2,655 (27.0)	<0.001	0.999
	Tobacco use	268,528 (7.8)	3,028 (30.8)	<0.001	0.998

**Table 2:** Multivariate-adjusted analysis of complications in patients with and without opioid use disorder.

Complication	Nonopioid dependent	Opioid dependent	Odds ratio^a^	*p* value (multivariate)
Prosthetic joint infection	22,702 (0.7%)	190 (1.9%)	1.55 (1.23–1.94)	<0.001
Superficial surgical site infection	24,020 (0.7%)	216 (2.2%)	1.66 (1.34–2.05)	<0.001
Noninfectious wound complication	30,492 (0.9%)	209 (2.1%)	1.40 (1.12–1.76)	0.003
Prosthetic complication	36,874 (1.1%)	217 (2.2%)	1.37 (1.10–1.70)	0.005
Revision	33,965 (1.0%)	238 (2.4%)	1.47 (1.19–1.81)	<0.001
Postoperative pain	135,788 (4.0%)	1,405 (14.3%)	2.97 (2.72–3.23)	<0.001

^a^Odds ratio with 95% confidence interval obtained through multivariate regression on matched cohorts while controlling for patient and operative characteristics.

**Table 3:** Multivariate analysis of healthcare utilization-associated factors.

Complication		Nonopioid dependent^a^	Opioid dependent^a^	Beta coefficient or odds ratio^b^	*p* value (multivariate)
TKA	Nonhome discharge	621,157 (27.9%)	1,729 (31.0%)	1.47 (1.30–1.64)	<0.001
	90-day readmission	190,769 (8.6%)	823 (14.8%)	1.60 (1.43–1.79)	<0.001
	Length of stay (days)	3.43 ± 2.74	4.39 ± 4.22	+0.67 days (0.56–0.78)	<0.001
	Inpatient cost (USD)	$17,013 ± 8,923	$20,394 ± 11,067	+$2,522 (2,170–2,874)	<0.001
	Total 90-day cost (USD)	$18,250 ± 11,575	$22,873 ± 15,793	+$3,602 (3,138–4,065)	<0.001

THA	Nonhome discharge	327,408 (27.3%)	1,357 (31.9%)	1.40 (1.28–1.53)	<0.001
	90-day readmission	112,624 (9.4%)	777 (18.2%)	1.53 (1.43–1.79)	<0.001
	Length of stay (days)	3.48 ± 3.59	5.26 ± 9.40	+1.09 days (0.95–1.24)	<0.001
	Inpatient cost (USD)	$17,943 ± 9,799	$22,307 ± 15,385	+$2,924 (2,496–3,353)	<0.001
	Total 90-day cost (USD)	$19,603 ± 13,252	$26,198 ± 21,360	+$4,257 (3,593–4,920)	<0.001

^a^Presented as mean ± standard deviation for continuous variables or *n* (%) for categorical variables, and univariate values represent unmatched cohorts. ^b^Odds ratio or beta coefficient with 95% confidence interval obtained through multivariate regression on matched cohorts while controlling for patient and operative characteristics; beta represents an increase in days and dollars associated with an opioid use disorder.

## Data Availability

The data used for this study are publicly available for a fee at the following web address: https://www.hcup-us.ahrq.gov/tech_assist/centdist.jsp.
